# Exploring the economic impact of inappropriate antibiotic use: the case of upper respiratory tract infections in Ghana

**DOI:** 10.1186/s13756-022-01096-w

**Published:** 2022-04-01

**Authors:** Jip Janssen, Samuel Afari-Asiedu, Annelie Monnier, Martha Ali Abdulai, Theresa Tawiah, Heiman Wertheim, Rob Baltussen, Kwaku Poku Asante

**Affiliations:** 1 Department for Health Evidence, Radboud University Medical Centre, Nijmegen, The Netherlands; 2 Kintampo Health Research Centre, Research and Development Division, Ghana Health Service, Kintampo North Municipality, Bono East Region Ghana; 3 Department of Medical Microbiology, Radboud Centre for Infectious Diseases, Radboud University Medical Centre, Nijmegen, The Netherlands

**Keywords:** Antibiotic resistance, Inappropriate antibiotic use, Economic impact analysis, Upper respiratory tract infection, Low and middle-income countries, Ghana

## Abstract

**Background:**

Antibiotic consumption is increasing worldwide, particularly in low and middle-income countries (LMICs). Access to lifesaving antibiotics in LMICs is crucial while minimising inappropriate use. Studies assessing the economic impact of inappropriate antibiotic use in LMICs are lacking. We explored the economic impact of inappropriate antibiotic use using the example of upper respiratory tract infections (URIs) in Ghana, as part of the ABACUS (AntiBiotic ACcess and USe) project.

**Methods:**

A top-down, retrospective economic impact analysis of inappropriate antibiotic use for URIs was conducted. Two inappropriate antibiotic use situations were considered: (1) URIs treated with antibiotics, against recommendations from clinical guidelines; and (2) URIs that should have been treated with antibiotics according to clinical guidelines, but were not. The analysis included data collected in Ghana during the ABACUS project (household surveys and exit-interviews among consumers buying antibiotics), scientific literature and stakeholder consultations. Included cost types related to health care seeking behaviour for URIs. Additionally, cost saving projections were computed based on potential effects of future interventions that improve antibiotic use.

**Results:**

Health care costs related to inappropriate antibiotic use for URIs were estimated to be around 20 million (M) USD annually, including 18 M USD for situation 1 and 2 M USD for situation 2. Travel costs and lost income due to travel, together, were estimated to be around 44 M USD for situation 1 and 18 M USD for situation 2. Possible health care cost savings range from 2 to 12 M USD for situation 1 and from 0.2 to 1 M USD for situation 2.

**Conclusions:**

This study indicates that inappropriate antibiotic use leads to substantial economic costs in a LMIC setting that could have been prevented. We recommend investment in novel strategies to counter these unnecessary expenditures. As the projections indicate, this may result in considerable cost reductions. By tackling inappropriate use, progress can be made in combatting antibiotic resistance.

**Supplementary Information:**

The online version contains supplementary material available at 10.1186/s13756-022-01096-w.

## Background

Antibiotic resistance is an increasing global health emergency and leads to treatment failure of patients with infectious diseases [1]. An important driver of antibiotic resistance is inappropriate antibiotic use, which is prevalent in low and middle-income countries (LMICs) with a high disease burden and inadequate access to approved healthcare [2, 3]. In the context of our study, inappropriate antibiotic use is defined as either using antibiotics unnecessarily (no clinical indication for antibiotics) or not using antibiotics when needed according to clinical guidelines (there is a clinical indication for antibiotics, however they are not provided). Studies have shown that lack of knowledge and not being able to recognise antibiotics may play a considerable role in inappropriate use [4, 5]. Inappropriate antibiotic use also includes self-medication, also known as non-prescription antibiotic use. Non-prescribed dispensing of antibiotics was found to be a common practice among community drug retail outlets (CDROs), a collective term that includes community pharmacies, drug stores or shops, rural drug vendors, and accredited drug dispensing outlets, in several sub-Saharan African countries: 69% of non-prescription antibiotics requests resulted in supply of antibiotics [2, 3, 5, 6]. Worldwide, most antibiotics are used in LMIC community settings where the burdens of infectious diseases and drug resistance are highest. So, there is an urgent need for pragmatic interventions to reduce inappropriate antibiotic use.

Therefore, the AntiBiotic ACcess and USe (ABACUS) I project was conducted between 2016 and 2019 to explore community antibiotic access and consumption practices in Asia and Africa in order to identify targets that inform the design of interventions to improve antibiotic use [2, 3, 7]. Findings showed that to improve appropriate antibiotic use, tailored interventions are needed as each setting is different. Antibiotics were confused with other medicines with a similar appearance which can impact inappropriate antibiotic use [5]. The present study is part of the currently ongoing ABACUS II project, which builds upon these findings [8].

Globally, there were an estimated 4.95 million (M) deaths associated with bacterial antimicrobial resistance (AMR) in 2019, including 1.27 M deaths attributable to bacterial AMR [9]. At the regional level, the all-age death rate attributable to resistance was estimated to be highest in western sub-Saharan Africa, at 27.3 deaths per 100,000. At current resistance rates, the total GDP effect in Organisation for Economic Cooperation and Development (OECD) countries, accounting for increased healthcare expenditure, would amount to 2.9 trillion USD by 2050 [10]. The future AMR costs are potentially large, imposing major costs to the world economy [11]. However, very little is known about the potential economic impact of inappropriate antibiotic use specifically, in terms of people unnecessarily buying (incorrect) medication and unnecessary travel time. Information on this knowledge gap is key, next to its public health impact, to indicate the importance to policy makers. In addition, an economic impact analysis can lay the basis for a more advanced cost-effectiveness analysis which reveals the interventions related to inappropriate use that provide value for money and can best be implemented.

The objective of this study is to explore the economic impact of inappropriate antibiotic use in the community setting in Ghana, related to upper respiratory tract infections (URIs) treated with antibiotics. It hereby fills an existing gap, as it is the first to provide such estimates for Ghana or any LMIC setting, and helps developing the economic case for sustainable investment in combating antibiotic resistance [12]. The study also provides hypothesised cost saving projections to estimate the different potential effects of future efforts that reduce inappropriate antibiotic use.

## Methods

### Study design

This was a top-down, retrospective economic impact analysis focussing on inappropriate use of antibiotics for the treatment of URIs in the Republic of Ghana. We focused on URIs as this group of infectious diseases is an illustrative example for which inappropriate use in the community is common [13, 14]. In Ghana, self-medication with antibiotics is widespread: 36% [4]. We based the URI case definition and incidence on the Global Burden of Disease (GBD) study, that mapped the ICD-10 codes ‘J00-J06.9’ and ‘J36-J36.0’ to the condition [15]. This includes e.g. infections of the nose, nasal cavity, tonsils, pharynx, and larynx with the subglottic area of the trachea [16]. For this analysis, antibiotic use was limited to oral solid formulations (capsules and tablets), thus excluding intravenous and intramuscular formulations and suspensions. Base year for the economic analysis was 2020. We adjusted data, if necessary, to this base year using inflation rates and demographic projections [17–19]. We followed WHO guidelines on costing analysis by adopting a societal perspective and using the ingredient approach, i.e. separately reporting quantities and unit prices, and adhered to the Consolidated Health Economic Evaluation Reporting Standards (CHEERS) checklist (see Additional file 1) [20, 21].

In Ghana, people with URI symptoms generally seek care at CDROs. All CDRO visits for URIs that lead to treatment that is not in accordance with antibiotic use guidelines, were reasoned to result in one of the following two inappropriate antibiotic use situations:

- *Situation 1:* URIs that were treated with antibiotics, against recommendations from clinical guidelines as considered self-limiting.
- *Situation 2:* URIs that should have been treated with antibiotics according to clinical guidelines as considered bacterial and sufficiently severe, but were treated with a painkiller.

We performed a separate economic impact analysis for each inappropriate use situation. A detailed overview of the included variables, values, and the information sources used for the analyses is shown in Tables 1 and 2.

**Table 1:** Economic impact analysis inappropriate use situation 1: variables, values, sources, and calculations (2020, rounded)

	Variable	Value	Code	Source
URIs	URI incidence (M) in population Ghana (31 M)	61*	A	GBD study and population Ghana [19, 22]
	% of URIs for which care was sought at CDROs	65%	B	Online expert opinion survey (see Additional file 2)
	Number of URIs for which care was sought at CDROs (M). *Calculation: C* = *A* × *B*	40	C	
	% of URIs for which care was sought that were treated with antibiotics, against recommendations from clinical guidelines	49%	D	Online expert opinion survey (see Additional file 2) and literature [6, 23–25]
	Inappropriate use situation 1, number of occurrences (M). *Calculation: E* = *C* × *D*	19	E	
Antibiotics	Weighted average unit price per antibiotic (USD) *Based on set of most commonly provided antibiotics for URI symptoms*^*§*^*, in their most common formulation (capsule or tablet) and dose*	0.05	F	Customer exit-interviews (ABACUS I database, 2018) and NHIS Medicines List [26]
	Weighted average number of antibiotics provided for a full course	18	G	Customer exit-interviews (ABACUS I database, 2018)
Income	Average income, per day (USD)	13*	H	Labour Force Report 2015, Ghana Statistical Service [27]
	Average income, per minute (USD)	0.03*	I	Labour Force Report 2015, Ghana Statistical Service [27]
Travel	Average round-trip travel costs to reach pharmacy to seek care for URI (USD)	0.9*	J	Household surveys (ABACUS I database, 2018)
	Average round-trip travel time to reach pharmacy to seek care for URI (minutes)	42	K	Household surveys (ABACUS I database, 2018)

	Variable	Code	Calculation
Annual costs	Health care costs	HCC	= E × F × G
	Travel costs	TC	= E × J
	Lost income due to travel	LIT	= E × I × K
	Cumulative costs	CC	= HCC + TC + LIT
	Cumulative costs per capita	CCPC	= CC ÷ population Ghana [19]

URI, upper respiratory tract infection; M, million; GBD, Global Burden of Disease; CDROs, community drug retail outlets; ABACUS, AntiBiotic ACcess and USe; NHIS, National Health Insurance Scheme (Ghana)

^*^Original value not from base year 2020, adjusted to 2020 using inflation rates and demographic projections[17–19]

^§^Amoxicillin, amoxicillin/clavulanic acid, azithromycin, ciprofloxacin, sulfamethoxazole/trimethoprim, flucloxacillin, and phenoxymethylpenicillin

**Table 2:** Economic impact analysis inappropriate use situation 2: variables, values, sources, and calculations (2020, rounded)

	Variable	Value	Code	Source
URIs	URI incidence (M) in population Ghana (31 M)	61*	A	GBD study and population Ghana [19, 22]
	% of URIs for which care was sought at CDROs	65%	B	Online expert opinion survey (see Additional file 2)
	Number of URIs for which care was sought at CDROs (M). *Calculation: C* = *A* × *B*	40	C	
	% of URIs for which care was sought that should have been treated with antibiotics according to clinical guidelines, but were treated with a painkiller	21%	L	Online expert opinion survey (see Additional file 2)
	Inappropriate use situation 2, number of occurrences (M). *Calculation: M* = *C* × *L*	8	M	
Painkillers	Weighted average unit price per painkiller (USD) *Based on set of most commonly provided painkillers for this situation*^§^	0.01	N	ABACUS I literature and NHIS Medicines List [5, 26]
	Average number of painkillers taken per day	3	O	Medical literature [28–30]
	Average number of days the painkillers are taken	5	P	Customer exit-interviews (ABACUS I database, 2018)
Income	Average income, per day (USD)	13*	H	Labour Force Report 2015, Ghana Statistical Service [27]
	Average income, per minute (USD)	0.03*	I	Labour Force Report 2015, Ghana Statistical Service [27]
Travel	Average round-trip travel costs to reach pharmacy to seek care for URI (USD)	0.9*	J	Household surveys (ABACUS I database, 2018)
	Average round-trip travel time to reach pharmacy to seek care for URI (minutes)	42	K	Household surveys (ABACUS I database, 2018)

	Variable	Code	Calculation
Annual costs	Health care costs	HCC	= M × N × O × P
	Travel costs	TC	= M × J
	Lost income due to travel	LIT	= M × I × K
	Cumulative costs	CC	= HCC + TC + LIT
	Cumulative costs per capita	CCPC	= CC ÷ population Ghana [19]

URI, upper respiratory tract infection; M, million; GBD, Global Burden of Disease; CDROs, community drug retail outlets; ABACUS, AntiBiotic ACcess and USe; NHIS, National Health Insurance Scheme (Ghana)

*Original value not from base year 2020, adjusted to 2020 using inflation rates and demographic projections[17–19]

^§^Diclofenac, ibuprofen, and paracetamol

### Data collection

For the analysis, we primarily collected unpublished data from the former ABACUS I project, originating from customer exit-interviews (~ 2300 interviewees) and household surveys (~ 1100 households visited) conducted in the Kintampo North and South districts, and from a newly conducted online survey using SurveyMonkey Inc. (see Additional file 2). The Kintampo districts are situated within the forest-savannah transitional ecological zone in the Bono East Region of Ghana and have a population size of approximately 156,000 [2, 5]. The online survey was necessary to obtain data on the following variables: % of URIs for which care was sought at CDROs (code ‘B’), % of URIs for which care was sought that were treated with antibiotics, against recommendations from clinical guidelines (code ‘D’ in Table 1), and % of URIs for which care was sought that should have been treated with antibiotics according to clinical guidelines, but were treated with a painkiller (code ‘L’ in Table 2). We distributed the survey among health care workers and public health researchers working in the African region, recruited through the ABACUS network. Respondents were asked for expert opinions on the different variables. In total, we collected 23 survey responses in January 2021 with a majority of responses coming from Ghana (82%). Experts from Ethiopia, Mozambique and Nigeria, working at a regional level and with work experience in Ghana, provided the remainder of responses. In the survey, each question covered a different variable. For each question, we asked respondents to base their estimate on a set of ranges. For the questions on codes ‘B’, ‘D’, and ‘L’, the options were: < 20%, 20–40%, 40–60%, 60–80%, and > 80%. All questions also included a ‘don’t know’ option, in case a participant was unable to provide an estimate. In addition to the ABACUS I data and expert opinions, existing scientific literature and (governmental) reports were consulted to approximate specific variable values.

### Analysis

Cost data was recorded in Ghanaian cedi, adjusted to 2020 price levels where relevant and subsequently converted into United States dollars using the average annual 2020 exchange rate [31]. We entered and analysed the data in Microsoft Excel Office 365. Collected data did not allow for sex and/or gender specific cost calculations. After all online survey responses were collected, we determined an average estimate per variable. For this, the provided ranges were first converted into a single mid-point value: 10%, 30%, 50%, 70%, and 90% for the answer options < 20%, 20–40%, 40–60%, 60–80%, and > 80% respectively. Besides average estimates, we calculated the corresponding standard deviations (SDs) and 95% confidence intervals (CI) to measure the amount of dispersion in the survey responses. This resulted in the following outcomes: care was sought at CDROs for 65% (SD: 17%, 95% CI: 56%, 73%) of URIs, 49% (SD: 18%, 95% CI: 40%, 58%) of URIs for which care was sought were treated with antibiotics against recommendations from clinical guidelines, and 21% (SD: 13%, 95% CI: 14%, 28%) of URIs for which care was sought should have been treated with antibiotics according to clinical guidelines but were treated with a painkiller.

Upon completion of the dataset, we first combined all variables to calculate the annual health care costs, travel costs, lost income due to travel, cumulative costs, and cumulative costs per capita [19]. Second, we determined the cost drivers. Third, we made cost saving projections for both inappropriate use situations, based on hypothesised reductions in situation occurrences. Hereby we aim to estimate the different potential effects of future efforts that reduce inappropriate antibiotic use. In the projections, the values for codes ‘D’ and ‘L’ were reduced with various percentages (10%, 30%, 50%, and 70%), reflecting possible outcomes of future efforts. As a result, adjusted outcomes of the annual cost variables were calculated. Here, implementation costs were omitted.

## Results

### Inappropriate antibiotic use: annual costs and cost drivers

The annual costs for inappropriate use situations 1 and 2 are presented in Table 3. For situation 1, the annual cumulative costs (61 M USD) mainly consisted of lost income due to travel (27 M USD - 43%), health care costs (18 M USD - 29%) and, lastly, travel costs (17 M USD - 28%). Regarding situation 2, the annual cumulative costs (20 M USD) were also mostly accounted for by lost income due to travel (11 M USD - 56%), followed by travel costs (7 M USD - 36%) and, lastly, health care costs (2 M USD - 8%).

**Table 3:** Economic impact analyses inappropriate use situations 1 and 2: outcomes (2020, rounded)

	Inappropriate use situation 1			Inappropriate use situation 2		
	Variable	Value (M USD)	% of CC	Variable	Value (M USD)	% of CC
Annual costs	Health care costs	18	29	Health care costs	2	8
	Travel costs	17	28	Travel costs	7	36
	Lost income due to travel	27	43	Lost income due to travel	11	56
	Cumulative costs (CC)	61		Cumulative costs (CC)	20	
	Cumulative costs per capita*	1.98		Cumulative costs per capita*	0.65	

M, million

*Population Ghana in 2020: 31 M [19]

### Cost saving projections

Different cost saving projections for both situations are presented in Table 4, based on hypothesised reductions in situation occurrences due to future efforts that reduce inappropriate antibiotic use. According to these projections, health care cost savings are higher for situation 1 (ranging from 2 M USD in case of a reduction of 10% to 12 M USD when a reduction of 70% is achieved) than for 2 (ranging from 0.2 to 1 M USD in case of a reduction of 10% and 70%, respectively). Savings related to reduced travel costs and reduced lost income due to travel are somewhat higher than health care cost savings for both situations.

**Table 4:** Potential effects of reducing inappropriate antibiotic use: cost saving projections (rounded)

	Inappropriate use situation 1. Projected reduction rates and associated cost savings (M, annually)					Inappropriate use situation 2. Projected reduction rates and associated cost savings (M, annually)				
	− 0% (baseline*)	− 10%	− 30%	− 50%	− 70%	− 0% (baseline*)	− 10%	− 30%	− 50%	− 70%
Number of situation occurrences	19	17	14	10	6	8	7	6	4	2
Health care cost savings (USD)	0	2	5	9	12	0	0.2	0.5	0.8	1
Travel cost savings (USD)	0	2	5	9	12	0	0.7	2	4	5
Lost income due to travel savings (USD)	0	3	8	13	19	0	1	3	6	8
Cumulative cost savings (USD)	0	6	18	31	43	0	2	6	10	14

M, million

*As presented in Tables 1 and 2, respectively

## Discussion

Our analysis suggests that inappropriate use of antibiotics has a considerable economic impact. For Ghana, related health care costs were estimated at around 20 M USD annually including 18 M USD for situation 1 and 2 M USD for situation 2. In addition, travel costs and lost income due to travel, together, were estimated to be around 44 M USD for situation 1 and 18 M USD for situation 2. A similar study reported substantial costs due to inappropriate antibiotic prescriptions for URIs of 297 M USD in Japan in 2016, yet estimates are not readily comparable because of differences in population size, URI epidemiology, and methodology [32]. Possible future health care cost savings range from 2 to 12 M USD for situation 1 and from 0.2 to 1 M USD for situation 2. This indicates that by reducing inappropriate antibiotic use in the future, a positive economic impact can be achieved at national level.

This study provides information that fills a long-standing knowledge gap in the field of antibiotic misuse. It is the first to explore the total economic costs involved, by addressing the complex and multiple components. Also, it indicates uncertainties in key variables such as the percentage of URIs inappropriately treated with antibiotics and has initiated data collection on these. Cost analysis literature so far usually focussed on assessing the economic impact of AMR, a stewardship activity in a specific care setting (e.g. an educational programme in a hospital department), or the use of diagnostics [33–36].

Understanding the economic consequences of community antibiotic consumption practices is crucial to mobilise key stakeholders and design sustainable strategies to improve antibiotic use. This economic impact analysis supports this endeavour by providing baseline data to be used by future studies investigating the cost-effectiveness of such strategies. In a broader sense, the study can serve as a starting point for other countries, especially LMICs, to conduct an economic analysis related to inappropriate antibiotic use.

The study has several limitations. First, it must be noted that inappropriate antibiotic use is a highly complex and comprehensive issue and providing a potential intervention is out of this study’s scope. Multiple factors can lead to URIs being treated in discordance with clinical guidelines. According to Godman et al. [37], sub-optimal management of URIs in LMICs, including the overuse of antibiotics, is promoted by misconceptions, social-cultural issues, diagnostic uncertainty, and clinical competency as well as commercial, patient, and time pressures. So, all these factors should ideally be considered when developing policy to tackle inappropriate use. Second, based on expert opinion we assumed that during all occurrences of inappropriate use situation 2, URIs were treated with painkillers. However, some URIs might have been treated with different medicine types or none at all (‘watch-and-wait’ approach). For these cases, the estimated health care costs may be inaccurate. Besides the cost saving projections, we did not carry out additional sensitivity analyses as cost estimates were largely linear with changes in input variables [38]. Third, in the absence of reliable data, the analysis did not include productivity losses due to inappropriate use of antibiotics. Involved costs are potentially large and future analysis should undertake efforts to collect data on e.g. number of extra days absent from work because of delayed antibiotic treatment for bacterial URIs. Notwithstanding these limitations, a strength of our study is the combination of various sources, i.e. expert opinion, previously unpublished data, existing literature, and (governmental) reports, to explore the economic impact of URIs.

## Conclusions

This study demonstrates that inappropriate antibiotic use leads to substantial economic costs in Ghana. These costs should be seen as unnecessary and preventable. We recommend investment in novel strategies to counter these expenditures. These interventions should be implemented in consultation with funders, industry, health care workers, pharmacists, policy makers, regulators, social scientists, and marketeers to ensure a successful roll-out. As the projections indicate, this may result in cost reductions. In addition, by tackling the inappropriate use problem, important progress can be made in combatting antibiotic resistance.

## Supplementary Information

**Additional file 1**. CHEERS checklist.

**Additional file 2**. Expert survey - questions and responses.

## Data Availability

The datasets used and/or analysed during the current study are available from the corresponding author on reasonable request.

## Abbreviations

LMICs: Low and middle-income countries
URIs: Upper respiratory tract infections
ABACUS: AntiBiotic ACcess and USe
M: Million
CDROs: Community drug retail outlets
AMR: Antimicrobial resistance
GBD: Global Burden of Disease
NHIS: National Health Insurance Scheme
SDs: Standard deviations
